# Pregnancy Close to the Edge: An Immunosuppressive Infiltrate in the Chorionic Plate of Placentas from Uncomplicated Egg Cell Donation

**DOI:** 10.1371/journal.pone.0032347

**Published:** 2012-03-27

**Authors:** Dorrith Schonkeren, Godelieve Swings, Drucilla Roberts, Frans Claas, Emile de Heer, Sicco Scherjon

**Affiliations:** 1 Department of Obstetrics, Leiden University Medical Center, Leiden, The Netherlands; 2 Department of Pathology, Massachusetts General Hospital, Boston, Massachusetts, United States of America; 3 Department of Immunohematology and Blood Transfusion, Leiden University Medical Center, Leiden, The Netherlands; 4 Department of Pathology, Leiden University Medical Center, Leiden, The Netherlands; Institut Jacques Monod, France

## Abstract

In pregnancies achieved after egg donation (ED) tolerance towards a completely allogeneic fetus is mediated by several complex immunoregulatory mechanisms, of which numerous aspects are still unknown. A distinct lesion not described previously in the literature, was repeatedly found in the chorionic plate in a substantial portion of placentas from ED pregnancies, but never in placentas from normal term pregnancies. The aim of this study was to assess its origin and its cellular composition. The relation between the lesion, the clinical and histological parameters were assessed. In addition we investigated the relation with the number of HLA-mismatches and KIR genotype of mother and child.

In ten out of twenty-six (38.5%) placentas from ED pregnancies an inflammatory lesion was present in the chorionic plate. A significantly lower incidence of pre-eclampsia was found in the group with the lesion; 0% versus 45.5%. A significant relation was found between this lesion and the presence of intervillositis, chronic deciduitis, presence of plasma cells and fibrin deposition in the decidua. Fluorescent in situ hybridisation with X/Y-chromosome probes showed that the majority of cells present in the lesion are of maternal origin. The expression of the macrophage marker CD14+ and of the type 2 macrophage (M2) marker CD163+ was significantly higher in the lesion. The incidence of a fetal HLA-C2 genotype was significantly higher in cases with a lesion compared to the group without the lesion. In conclusion, a striking relationship was observed between the presence of a not previously described inflammatory lesion in the chorionic plate and the absence of pre-eclampsia in ED pregnancies. The lesion consists of mainly maternal cells with a higher expression of the macrophage marker CD14+ and the M2 marker CD163+. These findings suggest a protective immune mechanism which might contribute to the prevention of severe clinical complications like pre-eclampsia.

## Introduction

During pregnancy the maternal immune system does not reject the fetus even though allogeneic paternal HLA antigens of the fetus are recognized by the maternal immune system. Tolerance to the semi-allogeneic fetus is mediated by several complex immunoregulatory mechanisms, of which numerous aspects are still poorly understood [1]. Immunologically even more intriguing are pregnancies achieved after egg donation (ED) in which the maternal immune system must maintain tolerance towards an often completely allogeneic fetus.

The increased risk of pre-eclampsia [2], [3] and a higher incidence of placental pathological lesions, such as chronic villitis of unknown etiology, chronic deciduitis and massive chronic intervillositis, found in ED cases is probably based on a dysregulation of the local immune response at the fetal-maternal interface [4], [5].

Decidual macrophages are thought to play an important regulatory role at the fetal-maternal interface. Decidual macrophages from normal pregnancies were molecularly characterized as M2 phenotype [6]. M2 macrophages are involved in tissue remodeling and inhibit inflammation [7] and therefore believed to be important for the maintenance of the tolerance to the non-self semi-allogeneic fetus [8].

The expression of Human leukocyte antigen-C (HLA-C) on the extravillous trophoblast is considered to be of critical importance in the acceptance of the fetus. HLA-C can interact with activating KIR receptors on uterine natural killer (uNK) cells and thereby modulate the immune function of these uNK cells. Hiby showed that certain combinations of fetal HLA-C, especially HLA-C2 and maternal KIR genotype (AA) were at a higher risk of developing pre-eclampsia [9], [10] .The KIR AA genotype is supposed to be associated with inhibition of uNK cell function.

Several studies in human term pregnancies and murine studies have shown an important role for T cells in specific immune tolerance to fetal alloantigens [11]–[13]. In addition, there is evidence that decidual T cells can specifically be activated by fetal HLA-C [14]. In uncomplicated pregnancies we have previously shown an association between the number of fetal–maternal HLA-C mismatches and the induction of functional regulatory T cells [14].

Gundosan *et al* [15] observed an inflammatory infiltrate of T-cells and uNK cells in the decidua of ED pregnancies. A resemblance with host versus graft disease is suggested in which a maternal alloimmune reaction is triggered by fetal cells present at the human fetal–maternal interface.

In the current study we investigated the histopathological features of placentas from a large group of ED pregnancies. During histological evaluation of the chorionic plate a distinct lesion of inflammatory cells was repeatedly found in a substantial proportion of placentas from ED pregnancies but never in placentas from normal term pregnancies. To our knowledge such a lesion has not been described previously in the literature.

In this study we characterize the origin and nature of the cellular infiltrate in the lesion. In addition its association with the number of HLA mismatches, KIR genotypes of mother and child was investigated. Further studies on histological and clinical parameters suggest that this lesion may reflect a protective immune mechanism of the mother's immune system towards the completely allogeneic fetus.

## Methods

### Patients

Prospectively we studied pregnancies from women conceived by egg cell donation. From all pregnancies paired samples of decidua basalis, decidua parietalis and heparinised maternal peripheral blood and heparinised cord blood were obtained. Maternal peripheral blood samples were obtained either directly before or directly after delivery. Data of medical records of all patients were reviewed. Pre-eclampsia was defined according to the classification of International Society for the Study of Hypertension in Pregnancy [16] as new hypertension (systolic bloodpressure of ≥140 mmHg and/or diastolic bloodpressure of ≥90 mmHg) after 20 weeks of gestational age in combination with proteinuria (≥300 mg in 24 hr). Signed written informed consent was obtained from all women, and the study received approval by the Leiden University Medical Center Ethics Committee (P02-200).

### Histology

After delivery either by caesarean section or vaginally, all placentas (n = 26) were macroscopically examined according to a standard protocol. Tissue samples were taken from the basal plate of the placenta and the fetal membranes, with a minimum of three pre-defined locations: centrally, in the periphery and one in between these locations. Tissue samples were fixed in 4% formalin and then routinely processed to paraffin blocks. Serial sections (4 µm thick) were cut on coated slides and dried overnight at 37°C.

The tissue specimens were stained with hematoxylin and eosin (H&E) and examined blind by a single pathologist (DR) and scored for pathological signs of chorioamnionitis [17], chronic villitis, intervillositis, chronic deciduitis, ischemic changes or infarction, perivillous fibrin deposition [18], increased syncytial knots and accelerated maturity.

### Fluorescent in situ hybridisation (FISH)

In order to determine the origin of the cells in the chorionic plate we performed fluorescent in situ hybridization (FISH) to visualize the X- and Y-chromosome in placentas derived from a male neonate with and without the lesion. This analysis was performed with the assumption that the chorionic plate of a placenta from a male neonate would be completely consist of male cells.

Serial sections were cut from three ED placentas with the chorionic plate lesion and from three ED placentas without the lesion, all from male neonates. Slides were deparaffinized and hydrated via graded alcohols to distilled water at 37°C. Heat induced antigen-retrieval was performed for 60 minutes at 80°C with citrate buffer followed by rinsing in distilled water at 37°C, three times in 2× SSC at 37°C and in PBS at 37°C. Slides were then treated with 0.05% pepsin in 0.01 M HCl at 37°C for 10 minutes, washed in PBS, and finally dehydrated in graded ethanol series up to ethanol 100% and air-dried. The slides were incubated with CEP X/Y DNA probe (Vysis/Abbott Molecular) and denaturation was performed at 80°C for 4 minutes, followed by overnight hybridization at 37°C in a moist chamber. After hybridization, slides were rinsed three times in 2× SSC/0.1% Tween at 37°C, three times in 0.1× SSC at 60°C and once in TNT buffer at room temperature. Subsequently, slides were dehydrated in ethanol series up to ethanol 100%, air-dried and mounted with Vectashield anti-fade reagent containing 50 µg ml^−1^ DAPI (4′,6-diamidino-2-phenylindole) for counterstaining of DNA.

### Immunohistochemistry

To determine the cellular composition of the infiltrate, immunohistochemistry was performed to analyze a broad panel of membranous receptors and intra-cellular markers of immune cells. Slides were deparaffinized and hydrated via graded alcohols to distilled water. After endogenous peroxidase activity was blocked for 20 minutes by 3% H_2_O_2_ in water, immunohistochemical staining was performed using the manufactures protocol. Briefly, heat-induced antigen-retrieval was performed for 20 minutes in a microwave. The slides were cooled down for 20 minutes followed by wash steps with first water and secondly PBS. The sections were incubated for one hour with primary antibody in the pre-determined dilutions in PBS with 1% BSA as described in Table 1. As a control the primary antibody was replaced by PBS with 1% BSA. After washing three times in PBS the slides were incubated for 30 minutes with Envision, followed by another washing step and incubation for 5 minutes with diaminobenzidine (DAB+, DAKO cytomation). Stained specimens were counterstained with haematoxylin (Sigma).

**Table 1:** Antibodies.

Epitope	Antigen retrieval	Primary antibody, dilution and source	Secondary antibody
CD45	None	Mouse mAB, 1∶100, Dako # M0701	Anti-Mouse Envision*
CD3	Tris/EDTA†	Rabbit IgG, 1∶150, Abcam # ab828	Anti-Rabbit Envision
CD4	Tris/EDTA	Mouse mAB, 1∶50, Novocastra, clone 1F6	Anti-Mouse Envision
CD8	Tris/EDTA	Mouse mAB, 1∶100, Novocastra, clone 4b11	Anti-Mouse Envision
CD56	Tris/EDTA	Mouse mAB, 1∶30, Dako, clone 123C3	Anti-Mouse Envision
CD14	Tris/EDTA	Mouse mAB, 1∶200, Novocastra, clone 7	Anti-Mouse Envision
CD163	Tris/EDTA	Mouse mAB, 1∶20, Abcam, clone 10D6	Anti-Mouse Envision
Cytokeratin-7	Tris/EDTA	Mouse mAB, 1∶2000, Dako, clone OV-TL 12/30	Anti-Mouse Envision
SMA	Tris/EDTA	Mouse mAB, 1∶500, Progen, clone ASM-1	Anti-Mouse Envision

† Tris/EDTA: 0.1 mol/L (pH 9.0).

* Envision: Dako, North America Inc, USA (anti-mouse and anti-rabbit Envision HRP).

Tissue slides were reviewed and scored by two independent observers (DS and GS). In areas with chorionic plate lesion and in the control placentas without the lesion present deposition of immunohistochemical stainings was scored semi-quantitatively. On a 4 point scale, staining intensity was graded from no staining deposition (1) to low focal deposition (2), moderate focal deposition (3) to diffuse deposition (4).

### HLA and KIR genotyping

All mothers and children were molecularly typed at low resolution level for the loci HLA-A, HLA-B, HLA-C, HLADRB1 and HLA-DQB1 using the Sequence Specific Oligonucleotides (SSO) PCR technique as described previously [14]. HLA typing was performed at the national reference laboratory for histocompatibility testing (LUMC, The Netherlands). The number of fetal–maternal HLA matches and mismatches was determined on the basis of the 2-digit DNA typing. The HLA-C1 and HLA-C2 group of both mother and child was established using the HLA-C low resolution DNA typing for the presence of ser77asn80 (C1) and asn77lys80 (C2) in the DNA sequence [19]. The number of C1 and C2 alleles present in the mother but not in the child (‘missing-self’) was determined.

To characterize the KIR genes of all mothers and children we performed a real time PCR method that identifies the presence or absence of 16 KIR genes, as previously described [20]. The haplotype A or B group of mother and child was established on basis of the number of activating and inhibitory KIR genes present [19]. Group A haplotypes possess a single activating gene, KIR2DS4, as well as four inhibitory genes encoding proteins representing the main HLA class I specificities, KIR2DL1, KIR2DL3, KIR3DL1 and KIR3DL2. Group B haplotypes possess different combinations of KIR2DL5, KIR2DS1, KIR2DS2, KIR2DS3, KIR2DS5 and KIR3DS1 genes. The HLA-C1 and HLA-C2 group of both mother and child were analyzed in the context of the group A and B haplotype of the KIR genes.

### Statistics

Categorical variables were compared using Fisher's exact test or the chi-square test. To determine differences between two groups with numerical data we used the one-sample T test. All statistical analyses were performed using SPSS software, version 17.0. P values less than 0.05 were considered significant.

## Results

### Patient characteristics

Patient characteristics of the two ED groups of pregnancies, those with and without the lesion in the chorionic plate are listed in Table 2. A significantly lower incidence of pre-eclampsia was found in the group with the lesion in the chorionic plate, 0% versus 45.5% (p = 0.03). There were no significant differences in the other maternal parameters. The average maternal age was respectively 40,3 years and 40 years in the group with and without the lesion in the chorionic plate. No significant differences were found in gestational age and fetal clinical outcomes between the two groups. There were no clinical symptoms of infection in mother and neonates during pregnancy, delivery or postpartum.

**Table 2:** Patient characteristics of the two ED groups of pregnancies, those with and without the lesion in the chorionic plate.

Clinical Parameters		No lesion present		Lesion present		P
**Mother**	**Age (years)**	40.0	(27–49)	40.3	(35–45)	0.91
	**Gravidity**	2.2	(1–7)	2.7	(1–5)	0.50
	**Parity**	0.3	(0–2)	0.7	(0–2)	0.21
	**Miscarriages**	0.6	(0–3)	0.6	(0–1)	0.98
	**Highest diastole (mmHG)**	87.3	(70–105)	78.4	(60–90)	0.07
	**Percentage Pre-eclampsia**	45.5%		0%		0.03*
**Delivery**	**Gestational age (weeks)**	38+2	(33–42)	37+6	(27–41)	0.78
	**Percentage Caesarian section**	63.6%		66.7%		0.63
**Child**	**Percentage Male Gender**	50%		60%		0.46
	**Birth weight (gram)**	2744	(1433–4100)	3171	(1028–4130)	0.26
	**Placental weight (gram)**	550	(326–880)	592	(360–835)	0.58

Average (range).

* **p<0.05.**

### Histopathology

**Figure 1:**
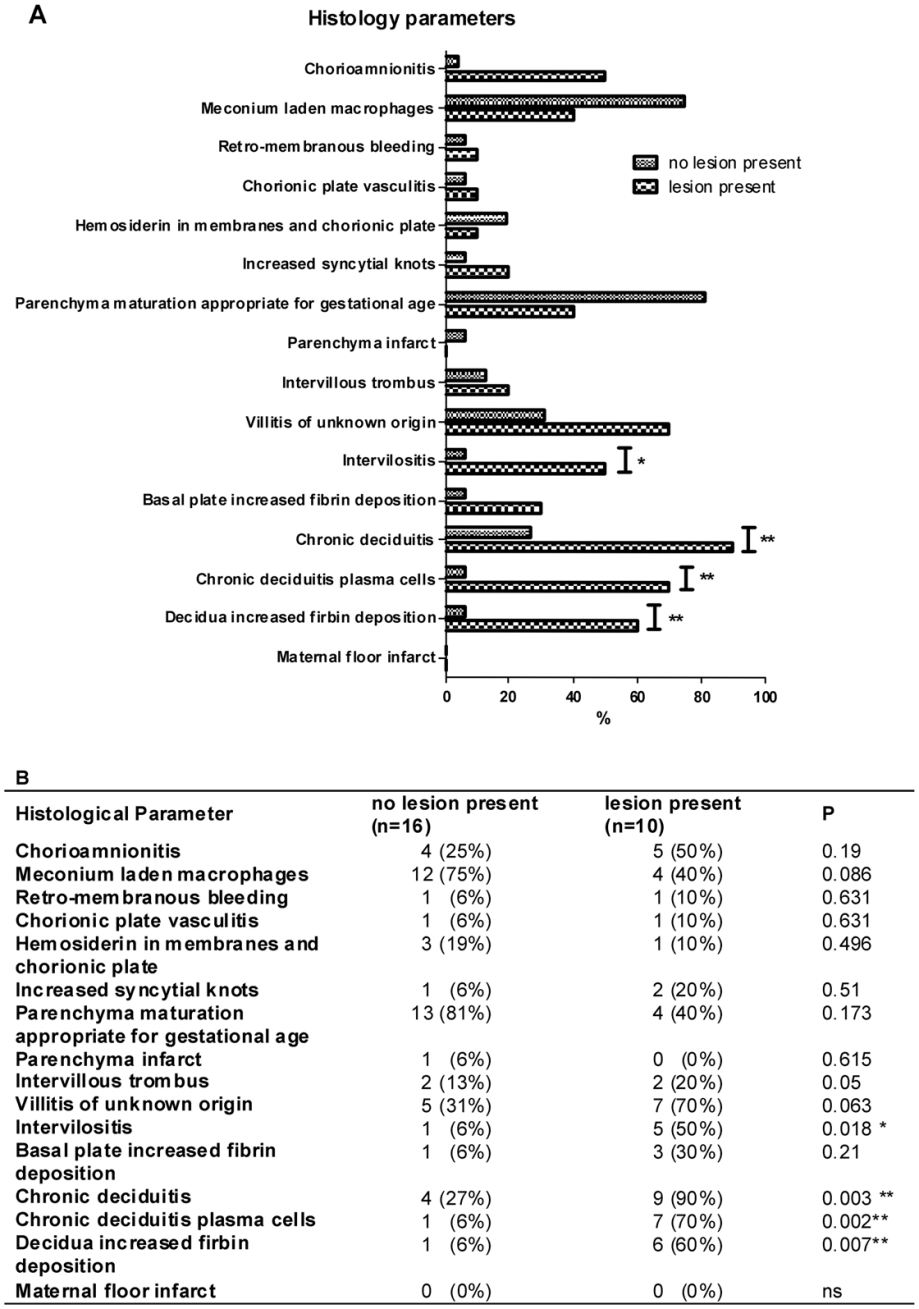
Significant relationship between the presence of the lesion in the chorionic plate and histological parameters. A. Bar graph of placental histological scores in percentage. B. Incidence of histological parameters in placentas with and without the lesion in the chorionic plate. A significant relationship was found between the presence of the lesion in the chorionic plate and intervillositis and histological parameters in the decidua; including chronic deciduitis, presence of plasma cells in the decidua and fibrin deposition in the decidua. Number (percentage) Fisher's exact test. * p<0.05 ** p<0.01.

In ten out of twenty-six placentas (38.5%) a lesion was present in the chorionic plate. The lesion was present in several locations in the chorionic plate and consisted of a diffuse inflammatory infiltrate involving the entire chorionic plate from the amniotic epithelium towards the intervillous space. We found an associated myxomatous change in the mesoderm of the plate and inflammatory cells within the plate and the subchorionic space. It was restricted to the chorionic plate and not on the chorion laeve. In the fetal membranes were no signs present of the lesion. No significant association was found between this lesion and the presence of chorio-amnionitis (Figure 1A–B). A significant relationship was found between the presence of the lesion in the chorionic plate and intervillositis (p = 0.018). Remarkably a significant association was found between the presence of this lesion and histological parameters in the decidua, including chronic deciduitis (p = 0.003), presence of plasma cells in the decidua (p = 0.002) and fibrin deposition in the decidua (p = 0.007) (Figure 1A–B). There was no significant association between the presence of the lesion in the chorionic plate and villitis of unknown origin (p = 0.063), or in signs of abnormal maturation.

### Origin of the infiltrate

The majority of cells present in the lesion in the chorionic plate comprise of cells with two X-chromosomes as detected by FISH analysis, which is compatible with an infiltrate by maternal cells. The maternal cell infiltrate was extended within the boundaries of the histological lesion.

**Figure 2:**
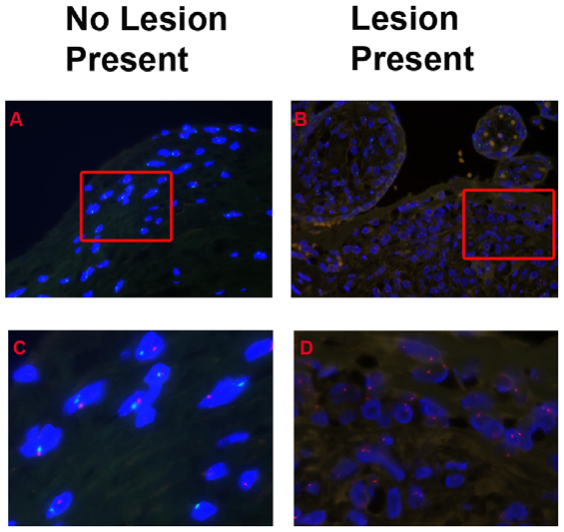
Majority of cells present in the lesion in the chorionic plate comprise of maternal cells. Fluorescent in situ hybridization of the X- and Y-chromosome in placentas derived from a male neonate, respectively red and green. Nuclei are stained blue. In the left panel (A,C) slides with no lesion present and in the right panel (B,D) slides with a lesion in the chorionic plate present. The lower panel (C,D) shows a magnification from the pictures in the upper panel. In the slides with lesion present all nuclei contain two red X-chromosomes.

In contrast we found in the chorionic plate of placentas without the lesion present a predominance of male XY-cells (Figure 2).

### Cellular composition of the infiltrate

**Figure 3:**
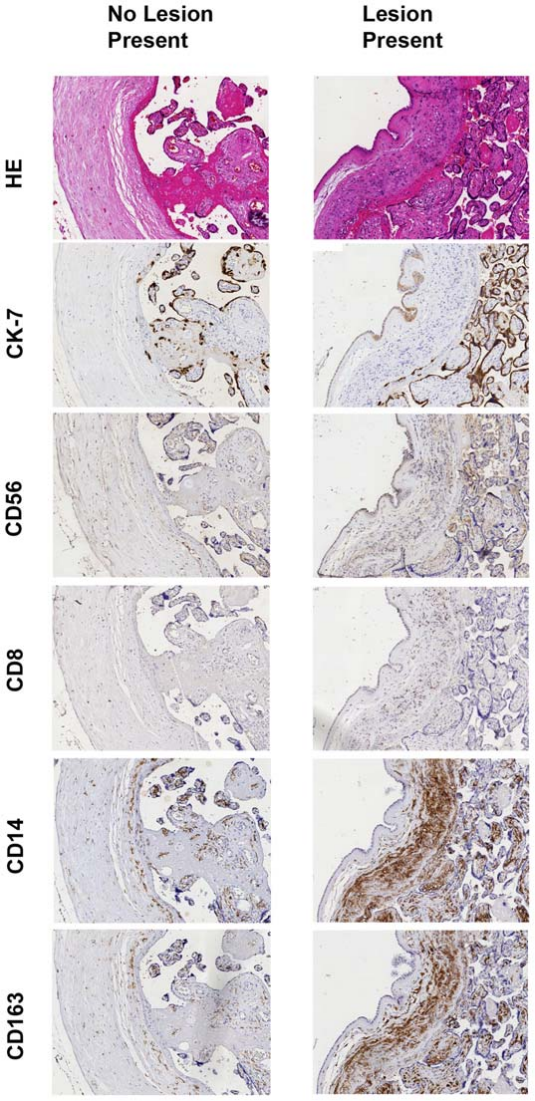
Higher expression of the macrophage marker CD14+, the M2 marker CD163+ and DC-Sign in the lesion. Immunohistochemical stainings of egg donation placentas. In the left panel serial slides with no lesion present and in the right panel serial slides with the lesion present in the chorionic plate. From top to bottom are depicted the stainings H&E, Cytokeratin-7, CD56+, CD8+, CD14+ and CD163+. We observed a higher expression of the macrophage marker CD14+ and the M2 marker CD163+.

Results of the immunohistochemical stainings are depicted in Figure 3. The expression of the macrophage markers CD14+ (p = 0.018) and macrophage scavenger receptor CD163+ (p = 0.018) was significantly higher in the group with the lesion present.

The T-cell markers CD3+, CD4+ and CD8+ showed no significant differences between the two groups. No correlation was found between the presence of the lesion and respectively the uNK cell marker CD56+ (Figure 3), the leukocyte marker CD45+ (data not shown), the fibroblast marker smooth muscle actin (data not shown) and the trophoblast marker Cytokeratine-7 (Figure 3).

### HLA+KIR typing

No correlations were found between the number of classical HLA-A, HLA-B, HLA-C, HLA-DRB1 and HLA-DQB1 mismatches between mother and child, and the presence of the lesion in the chorionic plate.

When the lesion in the chorionic plate is present, the incidence of a fetal HLA-C2 genotype is significantly higher (p = 0.037) compared to the group without the lesion (Table 3). No correlations were found between the HLA-C1 genotype of both mother and child in association with the lesion. When a fetal HLA-C2 genotype is combined with the maternal haplotype KIR B 66.7% of this group has the lesion present in the chorionic plate (Table 4). However this difference is statistically not significant. No other fetal HLA-C and maternal KIR haplotypes showed such a difference (Table 5).

**Table 3:** Number of fetal HLA-C2 genes present, in combination with the presence of the lesion in the chorionic plate.

Fetal HLA-C2 genes	No lesion present		Lesion present	
**No fetal HLA-C2 genes**	9	90.0%	1	10.0%
**One or two fetal HLA-C2 genes**	7	43.8%	9	56.3%

Number (percentage).

Fisher exact (2-sided) p = 0.037.

**Table 4:** Combination of fetal HLA-C2 and maternal KIR B, in comparison with the presence of the lesion in the chorionic plate.

Combination of fetal HLA-C2 genes with maternal KIR B haplotype	No lesion present		Lesion present	
**No combination of fetal HLA-C2/ maternal KIR B**	12	75.0%	4	25.0%
**Combination of fetal HLA-C2/ maternal KIR B**	3	33.3%	6	66.7%

Number (percentage).

Fisher exact (2-sided) p = 0.087.

**Table 5:** Combination of fetal HLA-C and maternal KIR haplotypes, in comparison with the presence of the lesion in the chorionic plate.

Combination of fetal HLA-C genes with maternal KIR haplotype	No lesion present	Lesion present	Fisher exact p value
**One or two fetal HLA-C1 genes/ maternal KIR AA**	5	1	0.34
**One or two fetal HLA-C1 genes/ one or two maternal KIR B**	8	6	1.00
**One or two fetal HLA-C2 genes/ maternal KIR AA**	3	3	0.65
**One or two fetal HLA-C2 gene/ one or two maternal KIR B**	3	6	0.087

Number.

Fisher exact (2-sided).

## Discussion

We observed a striking relationship between the presence of a not previously described lesion in the chorionic plate and the absence of pre-eclampsia, a relatively common complication in ED pregnancies [3]. In order to characterize cells possibly involved in the development of the lesion, we performed immunohistochemistry with several markers to phenotype the cells and *in situ* hybridization to determine their origin.

This report suggests an immunological protective mechanism in the chorionic plate of maternal cells directed against paternal antigens present in the fetal tissue. Intervillositis and several pathological parameters in the decidua are associated with this chronic lesion. Strikingly this lesion is absent in any of the cases complicated with pre-eclampsia. It is likely that this immunological activation might be a crucial factor in protection against pre-eclampsia .

Both the general macrophage marker CD14+ and the specific M2 marker CD163 were significantly upregulated in the placentas with the lesion. No definite relationship was found between the presence of the lesion in the chorionic plate and the expression of T-cell markers (CD3, CD4, CD8), NK-cell marker (CD56), trophoblast marker (cytokeratine-7) and myofibroblast expression (SMA). When the lesion in the chorionic plate is present then the fetal HLA-C2 genotype is significantly higher compared to the group without the lesion. Together these findings suggest a prominent role for an immunosuppressive macrophage subset in uneventful ED pregnancies,

Possibly the lesion in the chorionic plate is a distinct reaction of the mother's immune system to the often complete allogeneic fetus. The chorionic plate is never described before in normal physiological circumstances as a site of direct contact between maternal and fetal cells.

Gundogan *et al* [15] observed a lesion of chronic deciduitis with increased infiltration of CD4+ T-cells and CD56+ natural killer cells in the basal plate in egg donation pregnancies. No apparent involvement of macrophages were described in their studies. In contrast to Gundogan *et al* [15] we describe here a lesion found only in a group ED placentas infiltrated with macrophages at a different location, the chorionic plate. This lesion in the chorionic plate is associated with characteristic histo-pathological findings in the decidua.

The lesion was repeatedly found in the chorionic plate in a substantial portion of placentas from ED pregnancies, but never in placentas from non-egg donation pregnancies. We examined a group of 29 non-egg cell donation placentas of uncomplicated term pregnancies and a group of 25 non-egg cell donation placentas of pregnancies complicated by pre-eclampsia before 34 weeks of gestation and investigated the placentas for histological signs of a lesion in the chorionic plate. In neither of these two groups did we find a placenta with a lesion in the chorionic plate (data not shown).

Interestingly no effect on birth weight and neonatal outcomes were found between the group with and without the chorionic plate and lesion, regardless of the incidence of pre-eclampsia. More remarkable is our finding that pre-eclampsia only occurs in the group without the immunological lesion in the chorionic plate, regardless of the maternal age. Advanced maternal age is a well known risk factor for several pregnancy complications including pre-eclampsia [21]. Since increasing maternal age is nowadays the number one reason for ED, you would expect an association between ED pregnancies and pre-eclampsia. But even corrected for maternal age, pregnancies conceived with ED is an independent risk factor for pre-eclampsia.

The hypothesis that an unidentified cause of pre-eclampsia might include excessive or atypical maternal immune response to trophoblast is widely believed nowadays [22]. This would make pre-eclampsia a disease of failed interaction between two genetically different organisms [23]. Several genetic aspects have been described with a higher risk of pre-eclampsia. For example a limited sperm exposure with the same partner before conception increases the risk for pre-eclampsia [24], [25].

Paternal part of inheritance of the HLA could be an important factor of the maternal immune response to the fetal trophoblast. The trophoblast expresses only the HLA-C and HLA-G on its surface [26]. HLA-C antigens are the main ligands for the inhibitory KIRs, the specificity of the interaction being determined by a genetic polymorphism at positions 77 and 80.

Our study shows that the absence of a lesion in the chorionic plate is associated with pre-eclampsia, while there is a significantly lower incidence of the HLA-C2 genotype in the fetus. Hiby et all described that in conditions associated with defective placentation, mothers with a KIR AA genotype seem to more at risk when this genotype is combined with fetal HLA- C2 [9], [10] . This effect was true even if mothers had HLA-C2, indicating that neither non-self interactions nor missing self discrimination was a contributing factor. Moreover they described the protective effect of the presence of a KIR B region which reached significance when the fetus carried a HLA-C2 group [10]. This is consistent with our data that 66.7% of the group in which fetal HLA-C2 genotype is combined with the maternal haplotype KIR B has the protective lesion present in the chorionic plate. The fact that this difference is not statistically could be due to the small number of patients in this study.

However, our analysis was restricted to ED pregnancies that resulted in live births, which may have underscored the incidence of detrimental immune reactions in ED pregnancies resulting in spontaneous abortion.

This newly described lesion is probably not a separate entity, but part of a broad spectrum of histological pathology induced by the maternal immune system to the completely allogeneic fetus. Our results raise the possibility that the maternal immune system may exert a protective effect in preventing clinical complications like pre-eclampsia.
